# Healthcare workers as parents: attitudes toward vaccinating their children against pandemic influenza A/H1N1

**DOI:** 10.1186/1471-2458-10-596

**Published:** 2010-10-10

**Authors:** Sebahat D Torun, Fuat Torun, Binali Catak

**Affiliations:** 1 Assistt Rehberlik ve Müsteri Hizmetleri A.S., Istanbul, Turkey; 2 Umraniye Research and Training Hospital, Istanbul, Turkey; 3 Karabük Public Health Center, Turkey

## Abstract

**Background:**

Both the health care workers (HCWs) and children are target groups for pandemic influenza vaccination. The coverage of the target populations is an important determinant for impact of mass vaccination. The objective of this study is to determine the attitudes of HCWs as parents, toward vaccinating their children with pandemic influenza A/H1N1 vaccine.

**Methods:**

A cross-sectional questionnaire survey was conducted with health care workers (HCWs) in a public hospital during December 2009 in Istanbul. All persons employed in the hospital with or without a health-care occupation are accepted as HCW. The HCWs who are parents of children 6 months to 18 years of age were included in the study. Pearson's chi-square test and logistic regression analysis was applied for the statistical analyses.

**Results:**

A total of 389 HCWs who were parents of children aged 6 months-18 years participated study. Among all participants 27.0% (n = 105) reported that themselves had been vaccinated against pandemic influenza A/H1N1. Two third (66.1%) of the parents answered that they will not vaccinate their children, 21.1% already vaccinated and 12.9% were still undecided. Concern about side effect was most reported reason among who had been not vaccinated their children and among undecided parents. The second reason for refusing the pandemic vaccine was concerns efficacy of the vaccine. Media was the only source of information about pandemic influenza in nearly one third of HCWs. Agreement with vaccine safety, self receipt of pandemic influenza A/H1N1 vaccine, and trust in Ministry of Health were found to be associated with the positive attitude toward vaccinating their children against pandemic influenza A/H1N1.

**Conclusions:**

Persuading parents to accept a new vaccine seems not be easy even if they are HCWs. In order to overcome the barriers among HCWs related to pandemic vaccines, determination of their misinformation, attitudes and behaviors regarding the pandemic influenza vaccination is necessary. Efforts for orienting the HCWs to use evidence based scientific sources, rather than the media for information should be considered by the authorities.

## Background

Pandemic influenza A/H1N1 virus causes disease in all age groups, but it affects children stronger than adults in terms of attack rate and disease severity [1-3]. A study from United States reports that children were twice as susceptible to infection with the 2009 H1N1 virus from a household member as adults 19 to 50 years of age [4]. Nearly 30% of the first reported cases of pandemic influenza A/H1N1 in Turkey have involved persons who were 18 years of age or younger [5].

The social contact network, and the way people interact within, is critical to the spread of influenza [6]. Children are under greater risk for influenza infection because they experience a large number of extra-household contacts with their peers in daycare centers or schools and some of the most intense outbreaks have been associated with schools [7-9].

Healthcare workers have increased risk of getting infected with influenza during outbreaks because they are exposed to infected individuals in the community as well as hospitalized patients with influenza. They may become an important reservoir of influenza virus for vulnerable patients under their care [10-12] and especially for the children in their household [13]. Being a child of a HCW parent theoretically will increase the risk of influenza infection of the child, although no quantitative information is available on the subject. On the other hand it is reported that family members with influenza, especially children, rather than the daily burden of influenza patients, make every General Practitioner, vaccinated or not, very vulnerable to infectious influenza [14]. The role of children as the main sources of influenza transmission within a community or household has been referred in many studies [15-17].

The most effective countermeasure against a pandemic virus will be a specific pandemic vaccine available for the whole population [18]. However the supply of vaccines will be limited at least during the beginning of the influenza pandemic. Therefore, prioritization in the administration of the limited vaccine supply has been one of the major components in pandemic preparedness. The World Health Organization (WHO) recommends that HCWs, children aged above 6 months with one of several chronic medical conditions and healthy children should be considered as priority groups for pandemic influenza [19,20].

But influenza vaccine acceptance by HCWs is low even in the setting of pandemics [21-24].

Following the recommendations of the WHO, Turkish public health authorities have decided to start a mass vaccination campaign to mitigate the transmission of the pandemic influenza A/H1N1. The MoH has purchased 8 million doses of pandemic vaccine. In the first phase of vaccination campaign, vaccination has been offered to health care providers, people between the ages of 6 months and 50 years with underlying conditions, healthy people between 6 months and 24 years of age, pregnant women and Haj pilgrims [25]. On November 2, 2009, the vaccination campaign started in the country and vaccination was free of charge [26]. Pandemic influenza vaccination was not mandatory. Mass vaccination of schoolchildren in schools has been planned to begin at December. Until that date parents could vaccinate their children in primary health care settings. Vaccination of children in schools depended on written parental consent. Because the ratio of parents who gave written consent for vaccination of their children in schools was very low all over the country, the MoH cancelled the mass vaccination in schools and schoolchildren continued to be vaccinated in primary health care settings [27].

The aim of this study was to examine the attitudes of hospital HCWs as parents of children aged 6 months to 18 years compliance with pandemic influenza A/H1N1 vaccine during pandemic alert phase 6 in Istanbul.

## Methods

### Study design and participants

This study was conducted at Umraniye Research and Training Hospital (URTH), Istanbul, Turkey from 7-22 December 2009. The study hospital, hospital workforce and the population of the study have been reported elsewhere [24]. The participants of the previous reported study made up the study population of this study. Among all the 718 participants of the reported study, 389 HCWs met the inclusion criteria of being a parent of a child 6 month to 18 years of age.

### Data Collection

Data was collected by a self- administered, anonymous questionnaire [Additional File 1] which the participants could complete in less than ten minutes. During the time when the previous study questionnaires were taken up, the questionnaire was delivered by hand directly to the participants and taken back by the second author who is one of the HCWs at the study hospital. The questionnaire consisted of questions regarding age, sex, marital status, occupation, years of work in health services, history of seasonal influenza vaccination in 2009, source of knowledge for pandemic influenza, perception of risk and seriousness of pandemic influenza, agreement with pandemic vaccine safety and efficacy, confidence to Ministry of Health (MoH) about pandemics, children 6 months to 5 years of age in the household, school children (6 to 18 years of age) in the household, whether respondents and their children are vaccinated with pandemic influenza H1N1 vaccine and if not reasons for refusing pandemic influenza vaccine. Occupation was stratified into four groups based primarily upon education and training: (1) doctor, (2) nurse, (3) professional support staff and (4) nonprofessional support staff.

A brief oral and written description of the aim of the study was given to all the participants and verbal consent was obtained from the participants. Completed questionnaires were collected from the participants by the researchers within following days.

At time of our study was planned the activities of all the Research Ethic Committees (REC) were stopped in Turkey because the Turkish Medical Association has filed an action on 23 February 2009 requesting the cancellation of some of the provisions of the Regulation on the grounds that they are not in conformity with the international agreements in which Turkey is bound by [28]. Judicial order of the Council of State on 13.11.1009 with issue 2009/3991 E was motion for stay [29]. Authorization of our study is taken from Istanbul Provincial Directorate of Health.

### Statistics

Data was analyzed by SPSS 10.0 version. Descriptive statistics were computed for the survey responses and demographic information. The main outcome variable was the parental attitude towards vaccinating their children against pandemic influenza A/H1N1 (vaccinated versus not vaccinated). By cross tabulations, we analyzed univariate associations between attitude towards vaccinating their children against pandemic influenza A/H1N1 and the following variables: sex, age, occupation, duration of employment in health care service, self-vaccination against pandemic influenza A/H1N1and receiving seasonal influenza vaccine in 2009, source of knowledge about pandemic influenza, how serious they thought is the pandemic, weather they trust to the information they receive from Ministry of Health (MoH), agreement with pandemic vaccine safety and efficacy and agreement with some other statements related to pandemic. Pearson's Chi-square test was used for statistical analysis.

Univariate associations between child's pandemic influenza A/H1N1 vaccination status and other variables were tested by logistic regression. All independent factors associated with the acceptance of pandemic influenza A/H1N1 vaccination in cross tabulations were subsequently introduced in a backward multivariate logistic model. All of the analyses were two tailed, and *p *values < 0.05 considered as significant.

## Results

Self- reported pandemic influenza A/H1N1 vaccine receipt was not statistically different (p > 0.05) in HCWs who were parents of children 6 months-18 years and the other interviewed HCWs (24.4% and 20.6% respectively). Because the particular purpose of this report was to evaluate the attitudes of HCWs towards vaccinating their children with pandemic influenza A/H1N1 vaccine, data of 389 HCWs who have children aged 6 months-18 years, was further analyzed. Among 389 of HCWs 57.3% were female; their mean age was 35.0 ± 7.0 years (range 20-56 years); median time of occupation in health care service 96.0 months (range 1- 420 months). Seventy seven percent of parents had children who were 6 month to 18 years of age, while 22.9% had children only children 6 month to 5 years of age. Doctors and nurses made up 50.1% (n = 195) of the respondents. Table 1 represents the characteristics of the participants and results of univariate analyzes. One-hundred and five (27.0%) of the participants reported that themselves have been vaccinated against pandemic influenza A/H1N1. Among all parents 22.1% (n = 82) already get vaccinated their children against pandemic influenza A/H1N1, 66.1% (n = 257) do not plan to get their children vaccinated and 12.9% (n = 50) were still undecided. Among parents who do not plan to get their children vaccinated against pandemic influenza A/H1N1 or who were undecided about vaccination, the main reasons given for refusal were concerns about vaccine safety (245/307, 79.8%), or the respondents did not believe in efficacy of vaccination (177/307, 57.7%). Twenty one (6.84%) parent reported their reason for refusing the vaccine as "just don't want to get vaccinate". Other reasons given for refusal of vaccination included because the child has had the seasonal influenza vaccination (22/307, 7.2%), "just don't want to get vaccinate the child" (21/307, 6.8%), and because the prime minister did not get the vaccine (4/307).

**Table 1:** Characteristics of the respondents by attitude towards vaccinating their children (Values are percentages of respondents)

		Attitude towards vaccinating their children			
**Characteristic of parents**	**Total** **(N = 389)**	**Vaccinated** **(N = 82)**	**Not vaccinated** **(N = 257)**	**Undecided** **(N = 50)**	**p value**

**Gender**					

Female	57.3	17.5	71.7	10.8	0.023

Male	42.7	25.9	58.4	15.7	

**Occupation**					

Doctor	27.5	32.7	55.1	12.1	0.004

Nurse	22.6	17.0	69.3	13.6	

Professional support staff	11.8	15.2	60.9	23.9	

Nonprofessional support staff	38.0	16.9	73.6	9.5	

**Years of work in health services**					

< 5	40.1	19.9	69.9	10.3	0.361

5-9	12.6	22.4	71.4	6.1	

10-14	15.7	21.3	63.9	14.8	

≥ 15	31.6	22.0	60.2	17.9	

**Received pandemic influenza vaccine**					

Yes	27.0	60.0	24.8	15.2	< 0.000

No	73.0	6.7	81.3	12.0	

Sources of information*					

WHO and/or CDC besides MoH	13.6	37.7	52.8	9.4	0.003

MoH	36.0	22.2	62.1	15.7	

Physicians (no any health authority)	19.0	23.0	62.2	14.8	

Only media (television/newspaper)	31.4	11.5	78.7	9.8	

**Pandemic influenza A/H1N1 vaccine is safe**					

Agree/strong agree	48.6	36.0	46.6	17.4	< 0.000

Disagree	55.6	7.0	84.5	8.5	

**Vaccine is effective in preventing pandemic influenza**					

Agree/strong agree	56.3	31.5	52.1	16.4	< 0.000

Disagree	43.7	7.7	84.1	8.2	

**Trust the declarations and suggestions of Ministry of Health**					

Yes	57.6	29.5	56.2	14.3	< 0.000

No	42.4	9.7	79.4	10.9	

*MoH: Ministry of Health, WHO: World Health Organization, CDC: Centers for Disease Control and Prevention

The most reported source of information about pandemic influenza was media (television and newspaper) (83.0%). Nearly one third of the participants (31.4%) reported the media (television and newspapers) as their only source of information about pandemic influenza A/H1N1. The other most reported sources were Turkish Ministry of Health (49.6%), colleagues/doctors (49.4%), World Health Organization (13.6%), educational seminars of local health authority of the city (11.1%) and Centers for Disease Control and Prevention (3.1%).

An important proportion (42.4%) of the parents reported that did not trust in the declarations and suggestions of Turkish Ministry of Health (MoH) about pandemic influenza. No association was found between trust in the MoH and occupation.

Univariate analysis showed that gender, occupation, parents' self vaccination against pandemic influenza, source of knowledge regarding pandemic influenza, agreement with pandemic vaccine safety, agreement with vaccine efficacy and trust in the declarations and suggestions of MoH were significantly associated with the parental attitude toward vaccinating their children against pandemic influenza A/H1N1 (Table 1).

Logistic regression analysis showed that agreement/strong agreement with safety of the pandemic influenza vaccine (OR = 2.351; 95%CI: 1.116 - 4.953) and self receipt of pandemic influenza vaccine (OR = 13.624; 95%CI: 7.140 - 25.993) were significantly associated with positive parental attitude to vaccinate their children with pandemic influenza vaccine (Table 2).

**Table 2:** Logistic Regression Analysis: Factors associated with vaccinating their children with pandemic influenza A/H1N1 vaccine

Factors	Odds Ratio	95% CI	p value
**Self receipt of pandemic influenza vaccine**			

Yes	13.624	7.140 - 25.993	0.025

No	1	[reference]	

**Agreement with pandemic influenza A/H1N1 vaccine safety**			

Agree/strong agree	2.351	1.116 - 4.953	0.000

Disagree	1	[reference]	

**Trust in the declarations and suggestions of Ministry of Health**			

Yes	1.885	0.917-3.874	0.085

No	1	[reference]	

## Discussion

Our study revealed a low (21.1%) pandemic influenza A/H1N1 vaccination rate among children whose parents are healthcare workers. A study conducted in Italy at WHO pandemic alert phase 6 reports that 12.8% of mothers would have their children vaccinated, whereas 44.4% would remain doubtful [30]. Although in our study, the vaccination rate among children whose parents were doctor was higher (32.7%) than the children whose parents were nurse or support staff, we can come to the conclusion that even doctors, who are the most educated HCWs, were resistant to vaccinating their children against pandemic influenza A/H1N1. A recent study reports high education of the parent, being a health care worker and perception of vaccine ineffectiveness as determinants of fully negative attitude towards vaccination [31].

Lack of confidence in the MoH among our study population was thought provoking. The no confidence probably can be explained with dissidence between the MoH as the main authority in the national pandemic plan and the Turkish prime minister (the prime minister of the country had declared on November that neither he nor any one from his family will get the pandemic vaccine). This situation had found an important place the national media [32]. Unjustified fears about the adverse effects of pandemic influenza A/H1N1 vaccine, among HCWs as well as the public, have certainly been influenced by the mixed messages coming from vaccine-resistant Prime Minister. Media seems to be an important source of information (and sometimes the only information source even among doctors and nurses) about pandemic influenza A/H1N1 in our study population. Media can be accepted as a primary source of information in the general public, but our study group was HCWs and half of them were doctors and nurses. We believe that this finding is needs special investigation because in our opinion evidence based scientific sources and health authorities instead of the media should the primary information source of health care professions.

HCWs play a critical role in disseminating accurate information about pandemic influenza A/H1N1 vaccine. Besides being the main persons for implementation of pandemic influenza A/H1N1 vaccine, the HCWs and their family members are recipients of the vaccine. Therefore believes and attitudes of HCWs towards pandemic influenza A/H1N1 vaccination, can play an important role in supporting or blocking the vaccination efforts. A negative attitude of a HCW towards vaccination may grow as a snowball. A negative attitude of a HCW as a parent of a school child or a day care child, towards pandemic influenza vaccination possibly may have negative impact on the decision of the other parents of the classmates and teachers. An online survey conducted by a research company in Turkey, reports that out of 8,600 respondents 95% had not been vaccinated against pandemic influenza A/H1N1. Eleven percent of negative respondents had consulted a doctor about the vaccine, and therefore argues in the negative opinions and 10% percent said they were influenced by unfavorable opinion of Prime Minister, who previously said he would not receive the vaccine, in choosing refusing of the vaccine [33].

Our study has some limitations, and the results should be interpreted with these limitations. First limitation is that it covers only one hospital. Another limitation is the lack of details (especially their parental status) for the HCWs that we could not been interviewed (223/941, 23.6%) mainly because of the shift work in the hospital. Therefore a selection bias could have occurred. Lack of details about the age of their children and the fact that children could have chronic underlying disease is another important limitation of our study. Self-reporting vaccine uptake of children might be a potential bias also. All these points might limit to generalize our study results. Nevertheless, our study gives important information about HCWs actual attitudes as parents towards vaccinating their children with pandemic influenza A/H1N1 vaccine because it was conducted at an advance of pandemic phase.

Despite some limitations, our survey could be a useful tool for decision makers to promote programs and campaigns aimed at informing and educating HCW as well as parents.

## Conclusion

This study supports that persuading parents to accept a new vaccine seems not be easy even if they are HCWs. In order to overcome the barriers for vaccination among HCWs, determination of their misinformation, attitudes and behaviors regarding the pandemic influenza vaccination seems to be essential. For effectiveness of mass vaccination campaigns coherence between the authorities is important. Efforts for orienting the HCWs (especially doctors and nurses) to use medical and evidence based scientific sources, rather than the media for information related to health should be considered by the authorities. Efforts should be made to inform HCWs regarding the benefits of vaccination and the potential health consequences of influenza illness for their patients, themselves, and their family members.

Educational campaigns concerning the HCWs should include evidence based and comprehensible information about possible adverse effects of the vaccine and their incidence.

## Competing interests

The authors declare that they have no competing interests.

## Authors' contributions

SDT designed the study, reviewed the literature, analyzed the data and was responsible for writing the manuscript. FT participated in the coordination of the study, collected and entered the data, helped to literature review. BC helped to draft the manuscript and to interpret the results. All of the authors read and approved the final manuscript.

## Pre-publication history

The pre-publication history for this paper can be accessed here:

http://www.biomedcentral.com/1471-2458/10/596/prepub

## Supplementary Material

Additional file 1 **Questionnaire**. The questionnaire which was the source of information utilized in this article is shown, translated from Turkish. Click here for file
