# Supplementary material for: Healthcare workers as parents: attitudes toward vaccinating their children against pandemic influenza A/H1N1
Source: BMC Public Health. 2010 Oct 10;10:596. doi: 10.1186/1471-2458-10-596 (PMC3091558; doi:10.1186/1471-2458-10-596)
Supplement: Additional file 1 — Questionnaire. The questionnaire which was the source of information utilized in this article is shown, translated from Turkish. [file 1471-2458-10-596-S1.DOC]

Healthcare Workers as parents: attitudes toward vaccinating their children against pandemic influenza A/H1N1

| Dear Participant;  The aim of this study is to determine the attitudes of Health Care Workers (HCW) towards vaccinating their children against pandemic influenza in Umraniye Research and Training Hospital.  The study has permission from Istanbul Provincial Directorate of Health (Protocol number: SG.B104ISM.4344643/3285 date: 21.12.2010) and is conducted under the cognizance of the hospital administration.  You are free to be a participant in the research project entitled “Healthcare workers as parents: attitudes toward vaccinating their children against pandemic influenza A/H1N1”. If at any time during the session, you feel unwilling to continue, you are free to leave the study without negative consequences.  Your responses will be kept strictly confidential. Your name will not be linked with the research material; therefore *please do not write your name*.  We are interested in attitudes of health care workers towards vaccinating their children in general- not any particular individual’s answer in particular.  Because your answers will affect the study results please ;   - Answer all the questions, - Answer the questions honestly.   Thanks for participating our study.  Dr. Fuat Torun  Psychiatry Clinic  Phone: (535) 8259297  [**fuattorun@yahoo.com**](mailto:fuattorun@yahoo.com) |
| --- |

|  | ***Please answer all questions*** |
| --- | --- |
|  | Please indicate to what level you agree or disagree with the statements below:   |  | Strongly agree | Agree | Disagree | Strongly disagree | | --- | --- | --- | --- | --- | | There is a severe pandemic situation worldwide and in our country. | □ | □ | □ | □ | | The media is over-exaggerating the pandemic situation | □ | □ | □ | □ | | Overall the information I have heard about pandemic influenza has been clear. | □ | □ | □ | □ | | Pandemic influenza A/ H1N1 vaccine is safe | □ | □ | □ | □ | | Vaccine is effective in preventing pandemic influenza | □ | □ | □ | □ | |
|  | From which source/sources do you receive information about the swine flu?  *(Please tick one or more than one option)*   | □ The website of Turkish Ministry of Health (www.grip.saglik.gov.tr) | | --- | | □ The website of World Health Organization (www.who.int) | | □ The website of Centers for Disease Control (www.cdc.gov) | | □ Colleagues/ physicians | | □ Articles in scientific journals | | □ Educational seminars of local health authority of the city | | □ Media (Newspapers/television etc.) | | □ Other (Please State):_________________________________________ | | □ None of them. I don’t need to be informed about the outbreak. | |
|  | Do you trust the information provided and the recommendations by the national health authorities (Ministry of Health etc.) about pandemic influenza situation?  □ Yes □No |
|  | Did you receive a seasonal flu vaccine this year? □ Yes □ No |
|  | Did you receive pandemic influenza vaccine? □ Yes □ No |
|  | Do you have children under the age of 18 living at home, or not?  □ Yes □ No (***please skip to 10^th^ question and continue*** ) |
|  | At which age group are your children? (please indicate all the age groups in which your children are)  □ < 6 months of age  □ 6 months to 5 years of age  □ 5 years to 18 years of age |
|  | Did you vaccinate your children against pandemic influenza?  □ Yes, I have already vaccinated them  □ No, I will not vaccinate them  □ I am undecided |
|  | What are your reasons for refusing the pandemic influenza vaccine?  (Please state all the reasons that disincentive you to vaccinate your child/children against pandemic influenza?)  □ I am concerned about vaccine adverse reactions  □ Vaccine does not provide sufficient protection for swine flu  □ Pandemic influenza is not as serious as for conducting such a mass vaccination campaign  □ Just didn’t, nor any other reason  □Other ( Please state) _____________________________________________________ |
|  | **Please provide your details below:**  (THIS INFORMATION WILL BE KEPT STRICTLY CONFIDENTIAL) |
|  | How old are you? :__________ years |
|  | What is your gender? □ Male □ Female |
|  | What is your marital status? □ Married □ Divorced □ Widowed □ Single |
|  | What is your job title?     | □ Physician | □ Social workers | | --- | --- | | □ Nurse | □ Laboratory personnel | | □ Technician (any) | □ Office/Administrator | | □ Pharmacist | □ Cleaner | | □ Dentists | □ Security officer | | □ Physical Therapist | □ Other (Please state):____________________ | | □ Nutritionist |  | |
|  | What is your duration of employment in health care service? _______ years |
